# Altered functional connectivity within default mode network after rupture of anterior communicating artery aneurysm

**DOI:** 10.3389/fnagi.2022.905453

**Published:** 2022-07-25

**Authors:** Fuxiang Chen, Yaqing Kang, Ting Yu, Yuanxiang Lin, Linsun Dai, Lianghong Yu, Dengliang Wang, Xi Sun, Dezhi Kang

**Affiliations:** ^1^Department of Neurosurgery, The First Affiliated Hospital, Neurosurgery Research Institute, Fujian Medical University, Fuzhou, China; ^2^Department of Neurosurgery, Binhai Branch of National Regional Medical Center, The First Affiliated Hospital, Fujian Medical University, Fuzhou, China; ^3^First Affiliated Hospital, Fujian Provincial Institutes of Brain Disorders and Brain Sciences, Fujian Medical University, Fuzhou, China; ^4^Department of Radiology, The First Affiliated Hospital of Fujian Medical University, Fuzhou, China; ^5^School of Information Engineering, Nanyang Institute of Technology, Nanyang, China

**Keywords:** anterior communicating artery aneurysm, subarachnoid hemorrhage, cognitive impairment, resting-state fMRI, functional connectivity, default mode network

## Abstract

**Background:**

Rupture of anterior communicating artery (ACoA) aneurysm often leads to cognitive impairment, especially memory complaints. The medial superior frontal gyrus (SFGmed), a node of the default mode network (DMN), has been extensively revealed to participate in various cognitive processes. However, the functional connectivity (FC) characteristics of SFGmed and its relationship with cognitive performance remain unknown after the rupture of the ACoA aneurysm.

**Methods:**

Resting-state functional MRI (fMRI) and cognitive assessment were acquired in 27 eligible patients and 20 controls. Seed-based FC between unilateral SFGmed and the rest of the brain was calculated separately, and then compared their intensity differences between the two groups. Furthermore, we analyzed the correlation between abnormal FC and cognitive function in patients with ruptured ACoA aneurysm.

**Results:**

Cognitive impairment was confirmed in 51.9% of the patients. Compared with the controls, patients suffering from ruptured ACoA aneurysm exhibited a similar FC decline between each side of SFGmed and predominant nodes within DMN, including the precuneus, angular gyrus, cingulate cortex, left hippocampus, left amygdala, left temporal pole (TPO), and left medial orbitofrontal cortex (mOFC). Besides, significantly decreased FC of left SFGmed and left insula, right middle temporal gyrus (MTG), as well as right mOFC, were also found. In addition, only enhanced insular connectivity with right SFGmed was determined, whereas increased FC of the left SFGmed was not observed. Correlation analyses showed that lower total cognitive performance or stronger subjective memory complaints were related to reduced connectivity in the SFGmed and several cortical regions such as the angular gyrus and middle cingulate cortex (MCC).

**Conclusion:**

Our results suggest that patients with ruptured ACoA aneurysm exist long-term cognitive impairment and intrinsic hypoconnectivity of cognition-related brain regions within DMN. Deactivation of DMN may be a potential neural mechanism leading to cognitive deficits in these patients.

## Introduction

Aneurysmal subarachnoid hemorrhage (aSAH) is a life-threatening cerebrovascular disease with high mortality and morbidity, and 40% of them are due to rupture of anterior communicating artery (ACoA) aneurysm (Weir et al., 2002). Long-term cognitive impairments have been reported in approximately half of the survivors suffering from ruptured ACoA aneurysm (Martinaud et al., 2009), throwing a heavy burden to patients and their families because of insufficient awareness and limited therapeutic strategies. Currently, functional brain network-directed neuromodulation has shown great promise in various cognitive disorders, including poststroke dementia (Liu et al., 2019; Gimbel et al., 2021; Kolskår et al., 2021; Clancy et al., 2022). However, the neural mechanisms of impaired cognitive function have not been fully elucidated in patients after the rupture of an ACoA aneurysm, which undoubtedly restricts future potential therapeutic applications.

In recent years, neuroimaging studies with respect to cognitive deficits after aSAH have drawn increasing attention, but almost concentrated on structural brain changes, especially subcortical white matter (Yeo et al., 2012; Jang and Kim, 2015; Fragata et al., 2017a,b, 2018). For example, by analyzing brain diffusion tensor imaging (DTI) data in acute and subacute stages of aSAH, Fragata et al. (2017a,b, 2018) found that DTI parameters can early predict the occurrence of delayed cerebral ischemia and functional outcome. There were also some studies that unravel the central mechanisms of brain injury *via* the acquisition of DTI in the chronic stage, including consciousness deficit (Jang and Kim, 2015), motor weakness (Yeo et al., 2012), and cranial nerve damage following aSAH (Seo et al., 2015). Additionally, in comparison with subjects suffering from an unruptured aneurysm, microstructural white matter abnormalities were determined in patients with aSAH and that was linked with their cognitive impairment at 3 months after ictus (Reijmer et al., 2018). Collectively, DTI findings of these aSAH studies provide a necessary structural basis for further functional network research.

Resting-state functional MRI (fMRI) is an important non-invasive technique for the evaluation of brain networks and demonstrated to reliably reflect the spontaneous neural activity of the human brain (Egorova et al., 2018). As a result, investigators are greatly interested in using resting-state fMRI to explore the neural mechanism of cognitive impairment and evaluate the therapeutic effect of neuromodulation (Siegel et al., 2016). It is generally known that working memory and executive function are most susceptible to being impaired in patients with aSAH. Pathogenesis has been investigated *via* resting-state functional connectivity (FC) in a few studies (Maher et al., 2015; Mikell et al., 2015; Su et al., 2018). Compared with healthy controls, the authors discovered that there is multiple seed-based FC strength decline in the aSAH group, including the left parahippocampal gyrus, the left inferior temporal gyrus, and the left thalamus. Besides, abnormal cerebral FC of patients was significantly correlated with their poor memory performance (Su et al., 2018). Another study concerning executive function demonstrated that cognition-impaired patients with aSAH exhibit increased frontoparietal connectivity (Maher et al., 2015). These studies shed light on the brain network mechanisms underlying cognitive dysfunction after aSAH to some extent. But notably, locations of ruptured aneurysm were highly heterogeneous in the abovementioned studies, which undoubtedly cause different patterns of structural brain damage and functional network changes from the start. Additionally, hydrocephalus and epileptic seizure are common complications secondary to the rupture of ACoA aneurysm that may also result in long-term cognitive decline (Taufique et al., 2016; Neifert et al., 2021). Hence, these major confounding factors should be considered for a more accurate understanding of the characteristic brain network changes associated with cognitive dysfunction in patients with aSAH and provide the most precise brain network-directed treatments in the future.

Spontaneous subarachnoid hemorrhage in the anterior interhemispheric cistern on CT images is an important radiographic feature for the diagnosis of the ruptured ACoA aneurysm. It is also a contributing factor leading to frontal cortex structure and function damage, especially disruption in the medial gyri, including the subcallosal gyrus, anterior cingulate gyrus, and rectal gyrus, which have been identified in previous anatomical studies and were demonstrated to be responsible for the later developed cognitive impairment involving extensive domains (Martinaud et al., 2009; Beeckmans et al., 2020; Mugikura et al., 2020). In addition, SFGmed lesions detected by structural MRI due to the rupture of ACoA aneurysm were also discovered in correlation with cognitive executive deficit and task coordination deficit (Martinaud et al., 2009). Furthermore, imaging studies have suggested that the medial superior frontal gyrus (SFGmed) plays an important role in a variety of cognitive processes (Pan et al., 2021). As reported in various neuropsychiatric disorders with cognitive deficits, abnormal functional brain connectivity of SFGmed with several cognition-related nodes within the default mode network (DMN) was discovered (Qiu et al., 2021; Xu et al., 2021), including precuneus, amygdala, and anterior and posterior cingulate gyrus (Pan et al., 2021; Nagahama et al., 1999; Xu et al., 2021). But as far as we know, SFGmed-based resting-state FC has not been investigated in patients with aSAH caused by ACoA aneurysm rupture.

Hence, we separately defined bilateral SFGmed as the region of interest (ROI) and then used seed-based resting-state FC analysis to explore the features of cognition-related brain network in patients with aSAH caused by the rupture of ACoA aneurysm. We hypothesized that the decline of SFGmed-based FC strength in patients with aSAH due to ruptured ACoA aneurysm as compared to healthy controls, and abnormalities of some network features were related to their cognitive performance.

## Materials and methods

### Subjects

We recruited patients with aSAH who were hospitalized in our department. The inclusion criteria were: (1) aSAH due to the rupture of ACoA aneurysm; (2) aSAH history of more than 6 months; (3) age range from 35 to 70 years old; and (4) cognitive function intact before the aSAH onset. The exclusion criteria included: (1) history of stroke or neuropsychiatric diseases; (2) occurrence of delayed cerebral ischemia or epileptic seizure during hospitalization; and (3) appearance of hydrocephalus secondary to aSAH. The severity of aSAH at admission was rated using the Hunt–Hess scale (1). Healthy controls matched for age, gender, and level of education were consecutively enrolled in the local community. All the participants were right-handed. This study was approved by the Local Ethics Committee of the First Affiliated Hospital of Fujian Medical University. All the participants provided written informed consent.

### Magnetic resonance imaging acquisition

All the MRI data were collected using the same 3.0 T Siemens Imaging Scanner (Siemens Medical Solutions, Germany). Foam padding and earplugs were used to restrict head motion and minimize scanner noise. Subjects were instructed to stay awake with their eyes closed and to think of nothing during resting-state fMRI acquisition. Functional data were obtained using an echo-planar imaging sequence with the following parameters: 50 slices, thickness/gap = 3.4/0 mm, repetition time (TR) = 3,000 ms, echo time (TE) = 30 ms, flip angle = 90°, field of view = 240 mm × 240 mm, matrix = 80 × 80, and voxel size = 3.0 mm^3^ × 3.0 mm^3^ × 3.4 mm^3^. A total of 205 time points were collected for each subject and the resting-state data were acquired over 10 min. The T1-weighted images were acquired in the following parameters: TR = 2,300 ms, TE = 3.09 ms, flip angle = 9°, 192 sagittal slices, thickness = 1 mm, spaced = 0.5 mm, acquisition matrix = 256 × 256, field of view = 256 mm × 240 mm, and voxel size = 1.0 mm^3^ × 1.0 mm^3^ × 1.0 mm^3^. In addition, all the participants were scanned with T2-weighted images to exclude morphological abnormalities.

### Neuropsychological assessment

A neuropsychological test was carried out after MRI acquisition by two researchers who were blinded to this study. As previously reported, the Subjective Memory Complaints Questionnaire (SMCQ) was adopted to measure subjective memory problems in general and daily living, and the SMCQ score of 6 or above was assigned as a diagnostic threshold (Kim et al., 2021). Besides, all the participants were instructed to accomplish the Montreal Cognitive Assessment (MoCA), consisting of several cognitive domains such as memory, attention, visuospatial, and executive functions. Cognitive impairment was defined as the MoCA total score of less than 26 according to previously reported (Wong et al., 2012).

### Data processing

All the fMRI data were processed using Data Processing Assistant for Resting-State fMRI (DPARSF).^1^ First, the fMRI data were preprocessed. The data preprocessing was referred to the previous research (Chao-Gan and Yu-Feng, 2010), including: (1) slice timing; (2) head motion correction; (3) normalizing to the Montreal Neurological Institute (MNI) space (voxel size: 2 mm^3^ × 2 mm^3^ × 2 mm^3^); (4) smoothing by 8 mm full width at half maximum; (5) linear detrending; (6) bandpass temporal filtering (0.01–0.08 Hz); and (7) regressing out nuisance covariates (Friston 24 head motion parameters, whiter matter signal, and cerebrospinal fluid signal).

Then, binary masks of left SFGmed and right SFGmed were chosen to set as seed regions. Time courses from all the voxels within each seed were averaged and used as reference time courses. The Pearson’s correlation coefficient was calculated between the time courses of each reference and voxel, and then underwent Fisher’s z-score transformation. FC maps of the two regions were established and then analyzed in SPM12 using the ANOVA model for calculating the difference between group-level functional maps. Brain regions were considered significant within a threshold of *p* < 0.05 after the false discovery rate corrected for multiple comparisons and cluster size > 50.

### Statistical analysis

Between-group statistics were performed by using the SPSS software version 20.0. Demographic and clinical characteristics differences between the groups were compared by using an independent two-sample *t*-test and the chi-squared test. For non-parametric data, the Mann–Whitney *U*-test was used. A two-tailed Pearson’s correlation analysis was used to obtain the correlations between the FC values of the significant brain regions and the MoCA/SMCQ scores. The statistical significance threshold was set to *p* < 0.05.

## Results

### Demographic and clinical characteristics of the participants

Twenty-seven patients after rupturing of ACoA aneurysm and 20 healthy controls were enrolled for fMRI scans and cognitive assessment between April 2020 and March 2021. One patient and one control were excluded due to excessive head motion. The demographic and clinical characteristics of the remaining subjects are shown in Table 1. The Hunt–Hess scale for the majority of patients (25/26) at admission was grade 1–3, and the size of the aneurysm was almost small than 10 mm. There was no significant difference in age, gender, or years of education between the two groups. In addition, the MoCA and SMCQ scores were lower in patients with aSAH than in healthy controls (*p* < 0.01). Moreover, cognitive impairment was found in 51.9% (14/27) of all the patients with ruptured ACoA aneurysm, and subjective memory complaints in 44.4% (12/27) of patients.

**TABLE 1:** Clinical characteristics of the patients with ruptured ACoA aneurysm and healthy controls.

	Patients with ruptured ACoA aneurysm	Healthy controls	*P*-value
Age (year)	57.3 ± 9.8	53.3 ± 7.2	0.12
Gender (male/female)	15/11	10/9	0.77
Education (years)	9.1 ± 4.3	9.4 ± 4.1	0.623
**Hunt-Hess on admission**			
1	7	−	
2	16	−	
3	2	−	
4	1	−	
**Size of aneurysm**			
≤5 mm	12	−	
5–10 mm	13	−	
>10 mm	1	−	
**Aneurysm treatment**			
Coiling	8	−	
Clipping	18	−	
Interval between aSAH and MRI acquirement (month)	23.9 ± 13.4	−	
MoCA	23.88 ± 5.37	29.42 ± 0.84	<0.01
SMCQ	4.16 ± 3.88	0.11 ± 0.32	<0.001

The values were represented with mean ± standard deviation. aSAH, aneurysmal subarachnoid hemorrhage; ACoA, anterior communicating artery; MRI, magnetic resonance imaging; MoCA, Montreal Cognitive Assessment; SMCQ, Subjective Memory Complaints Questionnaire.

### Comparisons of functional connectivity strength between the two groups

**FIGURE 1:**
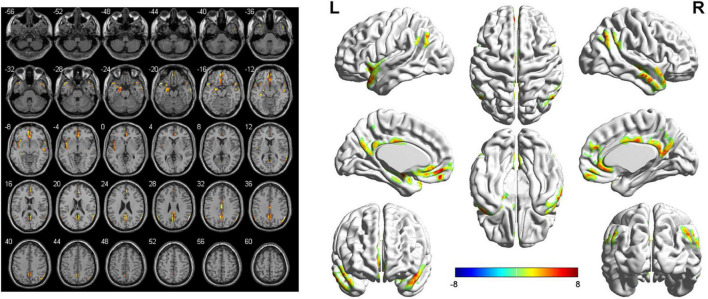
Left-side SFGmed-based resting-state functional connectivity analysis between patients with ruptured ACoA aneurysm and healthy controls. Results were displayed in 2D **(left)** and 3D **(right)**, respectively. The color bar represents T-scores. Brain regions labeling with color indicate decreased functional connectivity in patients with ruptured ACoA aneurysm as compared to the healthy controls. The threshold for displaying was set to *p* < 0.05, false discovery rate corrected, and cluster size > 50. Details of these color regions are given in Table 2. SFGmed, medial superior frontal gyrus; ACoA, anterior communicating artery; L, left; R, right.

**FIGURE 2:**
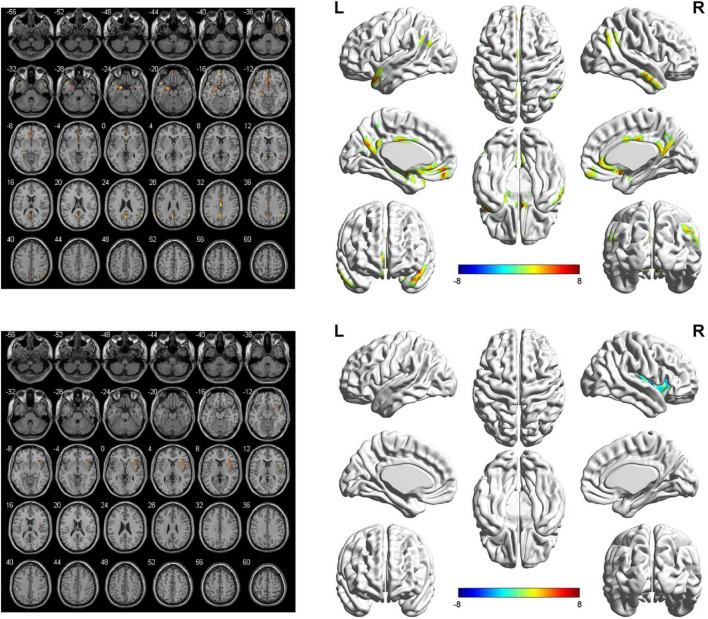
Right-side SFGmed-based resting-state functional connectivity analysis between patients with ruptured ACoA aneurysm and healthy controls. Results were represented in 2D (left) and 3D (right), respectively. The color bar represents T-scores. Brain regions labeling with color indicate decreased **(top panel)** or increased functional connectivity **(bottom panel)** in patients with ruptured ACoA aneurysm as compared to the healthy controls. The threshold for displaying was set to *p* < 0.05, false discovery rate corrected, and cluster size > 50. Details of these color regions are given in Table 2. SFGmed, medial superior frontal gyrus; ACoA, anterior communicating artery; L, left; R, right.

The seed-based FC analyses showed similar alternations in the brain network of bilateral SFGmed. Specifically, when the left SFGmed was defined as the ROI, a reduction in FC was found in the left temporal pole (TPO), left hippocampus, left amygdala, left insula, and right middle temporal gyrus (MTG), as well as the bilateral precuneus, bilateral angular gyrus, bilateral medial orbitofrontal cortex (mOFC), bilateral anterior cingulate cortex (ACC), bilateral middle cingulate cortex (MCC), and bilateral posterior cingulate cortex (PCC) in the aSAH group in comparison with the healthy controls. However, significantly increased functional brain connectivity of left SFGmed was not observed (Figure 1 and Table 2). Similarly, decreased functional brain connectivity in the bilateral precuneus, bilateral angular gyrus, and bilateral cingulate cortex was also uncovered when the right SFGmed was chosen as another ROI. In addition, we also found a decline in FC strength between the right SFGmed and left TPO, as well as right SFGmed and left mOFC. Conversely, compared with healthy controls, hyperconnectivity between right SFGmed and right insula was revealed in patients with ruptured ACoA aneurysm (Figure 2 and Table 3).

**TABLE 2:** Brain regions showing significant decreased left SFGmed-based functional connectivity in the aSAH group as compared to the healthy controls.

Brain region	Number of voxels	Peak MNI coordinates			Peak *T*-value
		X	Y	Z	
Right middle temporal gyrus	103	60	0	−26	8.13
Left temporal pole	127	−50	16	−20	8.99
Left hippocampus/left amygdala	235	−34	−30	−8	10.47
Left insula	192	−26	12	−22	8.27
Medial orbitofrontal cortex/anterior cingulate cortex	466	−4	52	−12	9.28
Right temporal pole	50	52	16	−18	5.25
Precuneus/posterior cingulate cortex	367	2	−44	24	7.87
Right angular	140	60	−58	28	11.91
Left angular	56	−50	−64	40	6.08
Middle cingulate cortex	99	2	−16	32	6.69

SFGmed, medial superior frontal gyrus; aSAH, aneurysmal subarachnoid hemorrhage; MNI, Montreal Neurological Institute.

**TABLE 3:** Brain regions showing significant differences of the right SFGmed-based functional connectivity in the aSAH group as compared to the healthy controls.

Brain region	Number of voxels	Peak MNI coordinates			Peak *T*-value
		X	Y	Z	
Left temporal pole	114	−50	14	−26	8.27
Left hippocampus/left amygdala	213	−34	−30	−8	8.38
Left medial orbitofrontal cortex	58	−4	50	−14	8.12
Anterior cingulate cortex	67	4	12	−16	6.96
Precuneus/posterior cingulate cortex	262	−2	−60	28	6.63
Right angular	93	60	−56	28	10.00
Left angular	54	−60	−56	30	6.90
Middle cingulate cortex	70	−2	−14	30	5.82
Right insula	138	40	0	0	−6.50

SFGmed, medial superior frontal gyrus; aSAH, aneurysmal subarachnoid hemorrhage; MNI, Montreal Neurological Institute.

### Correlations between functional connectivity strength and cognitive performance in the aneurysmal subarachnoid hemorrhage group

**FIGURE 3:**
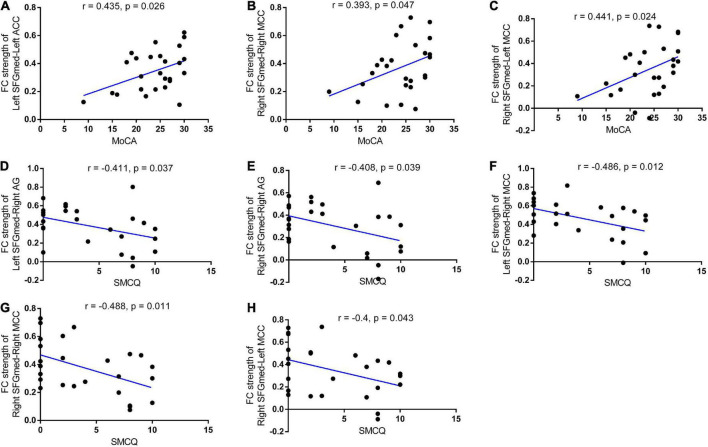
Correlations between functional connectivity strength and cognitive performance in patients with ruptured ACoA aneurysm. Positive correlations between the MoCA scores and left SFGmed-left ACC **(A)**, right SFGmed-right MCC **(B)**, as well as right SFGmed-left MCC **(C)** connectivity strength were discovered in patients with ruptured ACoA aneurysm. Negative correlations between the SMCQ scores and left SFGmed-right AG **(D)**, right SFGmed-right AG **(E)**, left SFGmed-right MCC **(F)**, right SFGmed-right MCC **(G)**, and right SFGmed-left MCC **(H)** connectivity strength were displayed in patients with ruptured ACoA aneurysm. ACoA, anterior communicating artery; SFGmed, medial superior frontal gyrus; AG, angular gyrus; MoCA, Montreal Cognitive Assessment; ACC, anterior cingulate cortex; MCC, middle cingulate cortex; SMCQ, Subjective Memory Complaints Questionnaire.

We also used correlation analysis to investigate whether the resting-state FC strength of the bilateral SFGmed seeds was associated with cognitive or memory performance. In patient with rupture of ACoA aneurysm, positive correlations between MoCA total score and left SFGmed-left ACC, right SFGmed-right MCC, as well as right SFGmed-left MCC FC strength were discovered (*r* = 0.435, *r* = 0.393, and *r* = 0.441, respectively). As shown in Figure 3, we also found negative correlations between the SMCQ scores and FC strength of right SFGmed-right MCC, right SFGmed-left MCC, and right SFGmed-right angular gyrus (*r* = −0.488, *r* = −0.4, and *r* = −0.408, respectively). In addition, the decreased FC strength between left SFGmed and right angular gyrus, as well as right MCC was significantly correlated with the increased score of the SMCQ (*r* = −0.411 and *r* = −0.486, respectively).

## Discussion

To the best of our knowledge, this is the first study to explore alterations in the resting-state functional brain connectivity of the SFGmed-based in patients with ruptured ACoA aneurysm as compared to the controls. The correlation between functional network changes and cognitive performance was also investigated. The current study primarily revealed decreased strength of FC between SFGmed and predominant brain regions within DMN, including the bilateral precuneus, bilateral angular gyrus, bilateral cingulate cortex, temporal cortex, and limbic system in patients after the rupture of ACoA aneurysm relative to healthy controls. Resting hypoconnectivity between some of the abovementioned brain regions such as SFGmed-right MCC, SFGmed-angular gyrus, and right SFGmed-left MCC was found to be significantly correlated with the MoCA and SMCQ scores, which have been widely demonstrated to participate in various cognitive deficits. Consistent with our hypothesis, our findings indicate a decline of extensive FC strength in cognition-related brain regions following the rupture of an ACoA aneurysm.

Cognitive dysfunction caused by neurodegenerative diseases has been an emphase by researchers around the world. In recent years, increasing attention has been paid to stroke-related cognitive deficits, which are mainly due to the high incidence of vascular stroke. Cognitive dysfunction is common in patients with aSAH due to ruptured ACoA aneurysm, no matter undergoing surgical clipping or endovascular coiling (Beeckmans et al., 2020). In this study, half (14/27) of the patients developed cognitive impairment approximately 2 years after the rupture of the ACoA aneurysm. Moreover, most of them belong to the Hunt–Hess scale grade of 1–3, i.e., low-grade aSAH. But conversely, another study involvement of 126 similar subjects existed only 22.2% of cognitive impairments (Ma et al., 2021). This discrepancy is likely due to the use of different cognitive assessment scales. In their study, the Modified Telephone Interview for Cognitive Status was adopted with the disadvantage of not being able to assess the cognitive function of subjects face-to-face, suggesting a possibility of subjective judgments. However, the present study employed a more widely used and valid MoCA scale (Wong et al., 2014). Subjective memory complaints were recognized as the most common domain of cognitive deficits described in patients with aSAH. Therefore, the SMCQ was also used in this study to assess subjective memory problems. Consistent with the results of the MoCA, subjective memory complaints were determined in 44.4% (12/27) of all the participants. On the other hand, the smaller size of patients with a ruptured history of ACoA aneurysm collected in our study may be another non-negligible factor.

Cognitive rehabilitation is necessary for many patients suffering from ruptured ACoA aneurysms, but the essential prerequisite is to grasp the exact neural mechanism of cognitive impairment. As reported in previous fMRI studies, abnormalities of frontoparietal executive network (Maher et al., 2015), frontal networks (Mikell et al., 2015), as well as the mirror neuron system (Plata-Bello et al., 2017) were discovered in patients with aSAH as compared to the control group. Besides, the altered connection properties were also associated with clinical dysfunction or cognitive manifestation. The distribution of aneurysms leading to aSAH varied in these studies, and even patients with intracranial hemorrhage were included. The homogeneity of patients is known to be importance for exploring the brain network mechanism of cognitive dysfunction, including the location of the ruptured aneurysm. The main advantage of this study is that only patients with ruptured ACoA aneurysm were included. What is more, confounding factors contributing to cognitive decline such as hydrocephalus and epileptic seizure were excluded. Therefore, the results of the present study may be more reliable to some extent.

Compared to the healthy controls, patients who survived the rupture of ACoA aneurysm exhibited a decline in resting-state FC between SFGmed and wide brain regions. Furthermore, the distribution of these cortical and subcortical brain regions with hypoconnectivity was basically similar, no matter which side of SFGmed was set as ROI, mainly including temporal cortex, mOFC, bilateral angular gyrus, bilateral cingulate cortex, bilateral precuneus, and limbic cortex (left hippocampus and left amygdala). Notably, the majority of these brain regions are known for vital components of the DMN, which is a well-established intrinsic large-scale brain network responsible for various cognitive processes such as recollection, imagination, semantic and episodic memory, and conceptual processing (Hsu et al., 2016; Smallwood et al., 2021). Substantial evidence has demonstrated that dysregulation, especially the deactivation of the DMN, is linked with many kinds of neuropsychiatric disorders, including stroke (Veldsman et al., 2018; Kolskår et al., 2021). In a matched case–control study, patients with poststroke cognitive impairment were observed to affect the DMN more frequently compared with controls (Lim et al., 2014). Structural evidence for DMN involvement comes from another study showing that correlations in the rate of atrophy within the DMN are more extensive after ischemic stroke (Veldsman et al., 2018). What is more, cognitive recovery in patients suffering stroke achieved from non-invasive treatment strategies was demonstrated to be associated with higher activation of nodes within DMN such as precuneus, PCC, temporal cortex, medial prefrontal gyrus, and angular gyrus (Batista et al., 2019; Kolskår et al., 2021). In addition, previous studies have suggested that the human DMN can be divided into two subsystems and a midline core region according to different responses to multifarious cognitive tasks. Among them, the medial temporal lobe subsystem consisting of the amygdala and hippocampus is linked with memory and emotional processes. Another subsystem mainly containing the temporoparietal cortex is associated with language and social cognition. Consistent with previous results, our findings of resting-state FC and correlation analyses are mainly located in the cognition-related subsystems of DMN (Robin et al., 2015; Hsu et al., 2016). Taken together, the decreased connection between nodes within the DMN is probably a central mechanism for cognitive impairment in patients with ruptured ACoA aneurysm.

Compared with healthy controls, the FC between SFGmed and insula was decreased in the left hemisphere, whereas increased in the right hemisphere in patients suffering from ruptured ACoA aneurysm. The insular cortex is involved in a variety of functions such as sensory stimuli and cognitive and emotional processing. Therefore, hypoconnectivity between the left SFGmed and left insular may be an intrinsic manifestation of cognitive impairment. The functional imaging studies have suggested that there exists abundant interinsular connectivity in physiological state. As a result, once the activity of left-sided insula is decreased, the contralateral insular cortex may play a compensatory role to limit the functional loss. This speculation is supported by a recent study of unilateral tumor infiltration of the insula whether dynamically modulates the FC of the contralesional one (Almairac et al., 2021). The potential functional plasticity in patients after a ruptured ACoA aneurysm also informs brain network-directed therapy.

There are several limitations to our study. First, cognitive function consists of multiple domains such as executive function, memory, attention, and visuospatial abilities. Although the MoCA and the SMCQ are widely considered suitable for the assessment of total cognitive impairment and subjective memory complaints, scales for other cognitive domains preferably also need to be evaluated. Second, the SFGmed can be separated into the supplementary motor areas and the pre-supplementary motor areas. Evidence suggested that they play important roles in motor and cognitive control, respectively. To better characterize the features of seed-based resting-state FC in patients after the rupture of the ACoA aneurysm, functional subdivisions of the SFGmed may be deserved. Third, we tried to minimize the effect of confounding factors on cognitive function, so the exclusion criteria for the present study included epileptic seizure during hospitalization and hydrocephalus at follow-up. In addition, we only recruited patients following rupture of ACoA aneurysm at our center in a relatively short period of time. As a result, a small group of eligible ACoA patients was included in our study. Studies with a larger sample size should be designed in the future to achieve a stronger conclusion.

In summary, the present study put forward evidence of a decline of FC between SFGmed and widespread brain regions in patients with ruptured ACoA aneurysm. Most of the cortical hypoconnectivity is mainly located in DMN, which has been correlated with poststroke cognitive deficits. Our findings implicate that decreased intrinsic connectivity within DMN may account for cognitive impairment following the rupture of ACoA aneurysm, which we hope will contribute to future translational therapy options after aSAH.

## Data availability statement

The original contributions presented in the study are included in the article/supplementary material, further inquiries can be directed to the corresponding author/s.

## Ethics statement

The studies involving human participants were reviewed and approved by the Local Ethics Committee of the First Affiliated Hospital of Fujian Medical University. The patients/participants provided their written informed consent to participate in this study.

## Author contributions

FC and DK conceived and designed the experiments. FC drafted the manuscript. DK revised the manuscript. YK performed the MRI scanning. TY and YL assessed the cognitive function. FC, DK, TY, YK, YL, LY, DW, and LD helped to collect patients and healthy controls. FC and XS participated in data processing and statistical analysis. All authors have read and approved the final version of the manuscript.

## Footnotes

1
http://www.restfmri.net
